# Functional Characterization of Fp2Cas9, a Cold-Adapted Type II-C CRISPR Nuclease from *Flavobacterium psychrophilum*

**DOI:** 10.3390/ijms262110681

**Published:** 2025-11-02

**Authors:** Ran Zhao, Jianqiang Zhu, Jing Wang, Di Wang, Xinting Liu, Lanlan Han, Shaowu Li

**Affiliations:** 1 Key Laboratory of Aquatic Animal Diseases and Immune Technology of Heilongjiang Province, Heilongjiang River Fisheries Research Institute, Chinese Academy of Fishery Sciences, Harbin 150070, China; zhaoran@hrfri.ac.cn (R.Z.); jianqiangzhu1998@163.com (J.Z.); wangjing@hrfri.ac.cn (J.W.); wangdi@hrfri.ac.cn (D.W.); 2 Department of Biosciences and Bioinformatics, School of Science, Xi’an Jiaotong-Liverpool University, Suzhou 215123, China; xinting.liu20@student.xjtlu.edu.cn (X.L.); lanlan.han@xjtlu.edu.cn (L.H.); 3 National and Local Joint Engineering Laboratory for Freshwater Fish Breeding, Heilongjiang River Fisheries Research Institute, Chinese Academy of Fishery Sciences, Harbin 150070, China

**Keywords:** Fp2Cas9, cold-adapted Cas9, *Flavobacterium psychrophilum*, PAM specificity, zebrafish genome editing

## Abstract

Cas9 with specialized temperature adaptations are essential for broadening the application of CRISPR-based genome editing across diverse biological contexts. Although Cas9 orthologs from thermophilic and mesophilic organisms have been characterized for high- and moderate-temperature applications, cold-active variants remain largely unexplored, limiting genome engineering in low-temperature systems such as aquaculture species. Here, we report the functional characterization of Fp2Cas9, a cold-adapted Type II-C nuclease from *Flavobacterium psychrophilum*. In vitro assays showed that Fp2Cas9 efficiently cleaves double-stranded DNA with a refined PAM requirement of 5′-SNAAAG-3′, and that its engineered sgRNA scaffold (sgRNA-V2) supports programmable DNA targeting. Notably, Fp2Cas9 retains 75% cleavage efficiency at 5 °C, approximately 2.5-fold higher than SpCas9 under the same conditions, but shows a marked reduction in activity at 35 °C. In vivo, a nuclear-localized variant (2NLS-Fp2Cas9) mediated efficient mutagenesis of the zebrafish *slc45a2* gene, yielding ~60% indel frequencies and pigmentation-deficient phenotypes in ~43% of injected embryos. Collectively, these findings establish Fp2Cas9 as a cold-adapted Cas9 with reliable activity at low temperatures. This work adds a valuable tool to the CRISPR-Cas9 toolkit and may facilitate genome editing in cold-water organisms and other low-temperature systems.

## 1. Introduction

Over the past decade, clustered regularly interspaced short palindromic repeat (CRISPR)-Cas systems have been established as adaptive immune mechanisms in prokaryotes, generating CRISPR RNAs (crRNAs) that target invasive genetic elements such as bacteriophages [1,2,3,4,5]. These systems confer immunity through three stages: adaptation (spacer acquisition into CRISPR arrays), expression (transcription and processing into mature crRNAs), and interference (crRNA-guided degradation of target sequences by Cas effectors) [6]. CRISPR-Cas systems are broadly divided by effector architecture: Class 1 employs multi-subunit complexes, whereas Class 2 relies on single-protein effectors [7]. Each class includes multiple types distinguished by their characteristic effector proteins, with Type II being the most abundant and well studied among Class 2 systems. The hallmark nuclease Cas9 mediates programmable DNA cleavage by associating with a crRNA and a tracrRNA, to recognize target sequences adjacent to a protospacer-adjacent motif (PAM) [8]. This molecular mechanism provides the foundation for modern CRISPR-Cas9 genome editing technologies.

A key breakthrough that enabled the widespread application of CRISPR-Cas9 technology was the development of the single-guide RNA (sgRNA). By fusing the targeting sequence of crRNA with the structural scaffold of tracrRNA, the sgRNA streamlined the system and allowed Cas9 to be programmed for site-specific genome editing [9]. Building on this advance, Zhang demonstrated that SpCas9 could induce precise double-strand breaks at endogenous loci in human and mouse cells, thereby establishing CRISPR-Cas9 as a practical genome-editing tool for mammalian systems [10]. Type II CRISPR-Cas systems, classified into subtypes II-A, II-B, and II-C [11,12], encompass a broad spectrum of Cas9 orthologs characterized from diverse bacterial species. While the widely adopted SpCas9 (II-A) demonstrated the power of CRISPR genome editing, its strict requirement for an NGG PAM emerged as a constraint, limiting targetable sites within complex genomes [9]. To address this limitation and expand the CRISPR toolkit, numerous other Cas9 orthologs belonging to these three subtypes have been characterized, including SuCas9 (*Streptococcus uberis*, NNAAA PAM) [13], SaCas9 (*Staphylococcus aureus*, NNGRRT PAM) [14], CjCas9 (*Campylobacter jejuni*, NNRNRYAC PAM) [15], NmCas9 (*Neisseria meningitidis*, NNNNGATT PAM) [16], AnoCas9 (*Anoxybacillus flavithermus*, NNNNCDAA PAM) [17], and St1Cas9 (*Streptococcus thermophilus*, NNRGAA PAM) [18], each with distinct PAM preferences. Notably, many Type II-C Cas9 nucleases recognize more complex PAM sequences, a property often associated with enhanced target specificity and reduced off-target activity [19,20].

Besides PAM compatibility, temperature is a critical factor that limits Cas9 functionality in practical applications. The optimal operating temperature of Cas9 directly influences cleavage efficiency, thereby influencing its applicability across different biological systems. Currently, the most widely used gene-editing tool, SpCas9, derived from mesophilic bacteria, exhibits peak activity at 37 °C, displaying high cleavage efficiency and specificity under standard mammalian cell culture conditions [21,22,23,24]. In contrast, certain thermophilic Cas9 homologs, including GeoCas9 and ThermoCas9, retain activity at temperatures up to 70 °C, thereby expanding the CRISPR toolkit for high-temperature applications [25,26]. However, cold-adapted Cas9 systems active at low temperatures remain uncharacterized, presenting a major limitation for genetic engineering in cold environments. This challenge is especially pronounced in aquaculture, where numerous economically important cold-water fish species (e.g., *Salmo salar*, *Oncorhynchus mykiss*) inhabit environments of approximately 5–20 °C [27,28]. Under these conditions, SpCas9 activity is substantially reduced, impeding efficient and precise genome editing [29]. Addressing this gap requires the discovery of Cas9 with cold-adapted properties. In previous work, we identified a type II-C Cas9, named Fp2Cas9, from *Flavobacterium psychrophilum* and confirmed its functionality via conjugation transfer [30]. However, the nuclease cleavage activity of Fp2Cas9 under low-temperature conditions and its application potential have not been validated through in vitro experiments.

Based on the previously described research background and current challenges, this study systematically characterized the function and low-temperature adaptation of Fp2Cas9 from *F. psychrophilum*. We used bioinformatic approaches to predict the evolutionary relationships, functional domains, and three-dimensional structure of Fp2Cas9. The Fp2Cas9 protein was then expressed and purified, with nuclear localization signals optimized for zebrafish embryo editing. Next, we systematically compared the in vitro DNA cleavage activities of Fp2Cas9 and SpCas9 across various temperatures. Finally, the editing efficiency of Fp2Cas9 in vivo was validated by targeting the *slc45a2* gene in zebrafish (*Danio rerio*), using both molecular and phenotypic analyses. This study provides the first comprehensive demonstration of the high efficiency of Fp2Cas9 at low temperatures and introduces a new tool for gene editing in low-temperature biological systems and aquaculture breeding, thereby expanding the potential applications of CRISPR-Cas9 technology under low-temperature conditions.

## 2. Results

### 2.1. Bioinformatic Characterization of Fp2Cas9

**Figure 1:**
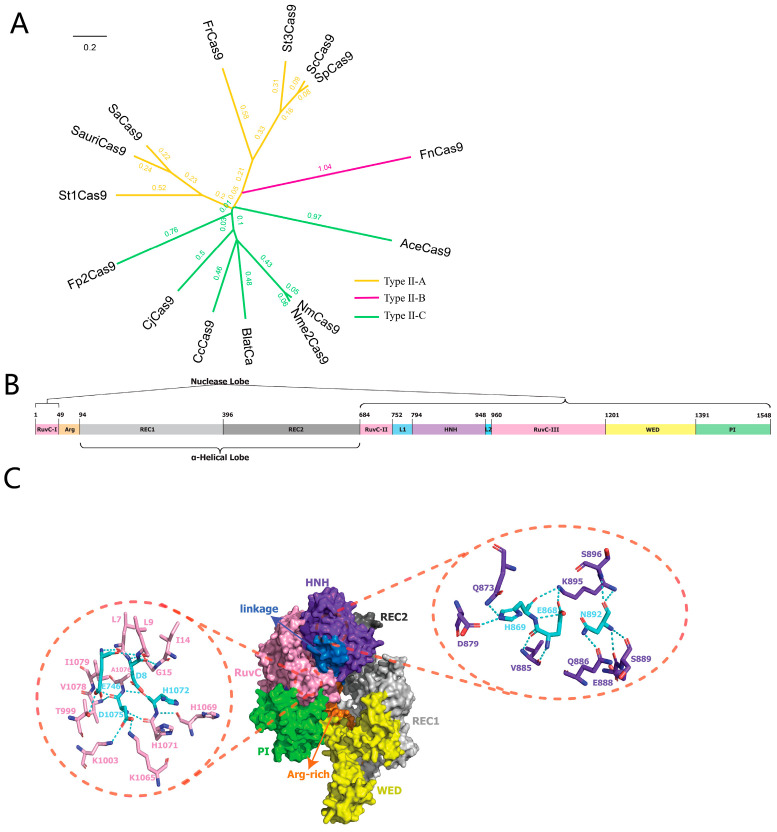
Bioinformatic analysis of the Fp2Cas9 nuclease. (**A**) Phylogenetic analysis of Fp2Cas9 based on BLASTp alignment. (**B**) Schematic of the Fp2Cas9 domain organization. (**C**) AlphaFold3-predicted structure of the Fp2Cas9 apo protein (ipTM = 0.84). Insets show the conserved catalytic residues in the HNH and RuvC nuclease domains. Key catalytic residues are highlighted in cyan. Dashed lines indicate predicted polar interactions. A nucleic acid binding channel is visible between the nuclease lobe (RuvC, HNH) and the α-helical lobe (REC domains). The HNH domain is positioned distally from the channel.

The Fp2Cas9 open reading frame spans 4647 nucleotides and encodes a protein of 1548 amino acids. To characterize Fp2Cas9 bioinformatically, we first conducted a phylogenetic analysis (Figure 1A). The analysis showed that Fp2Cas9 clusters within the Type II-C Cas9 subtype, exhibiting closer evolutionary relationships with homologs such as AceCas9, CjCas9, and NmeCas9, and more distant relationships with Type II-A (e.g., SpCas9, ScCas9) and Type II-B (FnCas9) enzymes. Domain architecture analysis indicated that Fp2Cas9 contains the characteristic domains of Cas9 nucleases, including the REC (recognition), HNH nuclease, and PI (PAM-interacting) domains, which are essential for DNA recognition and cleavage (Figure 1B). Structural analysis further revealed the polar interactions between catalytic residues and surrounding amino acids in these domains, offering insights into the structural basis of Fp2Cas9 nuclease activity (Figure 1C). Collectively, these analyses define the evolutionary, domain, and structural features of Fp2Cas9, providing a foundation for further investigation of its functional properties as a nuclease.

### 2.2. Characterization of Fp2Cas9 Nuclease

We previously validated the Type II-C CRISPR/Cas system of *F. psychrophilum* CN46 at the prokaryotic level, demonstrating its ability to cleave an exogenous plasmid containing spacer2 via conjugation experiments [30]. Building on this finding, we sought to characterize the Fp2Cas9 in vitro. The codon-optimized Fp2Cas9 gene was cloned into the pGEX-6P-1 vector (Table S1), expressed in *Escherichia coli* BL21 (DE3), and purified to >95% purity (Figure S1). Full-length tracrRNA and crRNA-sp2 sequences, predicted from previously reported sequencing data, were synthesized by in vitro transcription (Table S2).

**Figure 2:**
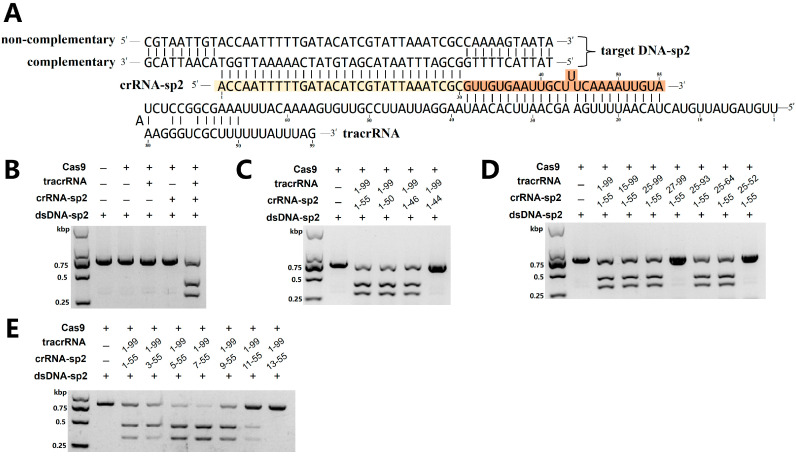
Functional analysis of Fp2Cas9 in vitro. (**A**) Schematic representation of tracrRNA, crRNA−sp2, and protospacer 2 DNA sequences. Regions of crRNA complementary to tracrRNA (orange) and the protospacer DNA (yellow) are indicated. (**B**) Fp2Cas9 complexed with a 55−nt crRNA−sp2 (containing spacer 2) and a 99−nt tracrRNA efficiently cleaved exogenous dsDNA containing the cognate protospacer 2 sequence. (M, DNA marker; kbp, kilobase pairs) (**C**) Cleavage assays using Fp2Cas9−tracrRNA: crRNA complexes reconstituted with 99−nt tracrRNA and scaffold−truncated crRNA−sp2 constructs. (**D**) Cleavage assays using Fp2Cas9−tracrRNA: crRNA complexes reconstituted with 55-nt crRNA−sp2 and truncated tracrRNA constructs. (**E**) Cleavage assays using Fp2Cas9−tracrRNA: crRNA complexes reconstituted with 99−nt tracrRNA and spacer−truncated crRNA-sp2 constructs. (kbp, kilobase pairs).

DNA cleavage assays confirmed that purified Fp2Cas9, when complexed with tracrRNA and crRNA-sp2 as illustrated in Figure 2A, efficiently cleaved exogenous double-stranded DNA (dsDNA) substrates (Figure 2B). To delineate the minimal functional regions of tracrRNA and crRNA-sp2, secondary structural features were predicted from sequence analysis (Figure 2A). Guided by these inferred structures, a series of truncation variants were systematically generated for both tracrRNA and crRNA-sp2. Cleavage assays with these truncated RNAs revealed that the core functional sequence of crRNA-sp2 lies within nucleotides 1–46 (Figure 2C), whereas the essential region of tracrRNA spans nucleotides 25–64 (Figure 2D).

We further investigated spacer length requirements by systematically evaluating variants of 18–30 nt on in vitro cleavage efficiency. Fp2Cas9 retained nuclease activity with spacers of 22–30 nt, exhibiting peak efficiency at 24–26 nt (Figure 2E). Collectively, these analyses define the structural and functional core of the crRNA and tracrRNA, providing a foundation for subsequent in vivo genome editing applications.

### 2.3. Fp2Cas9 In Vitro PAM Analysis

**Figure 3:**
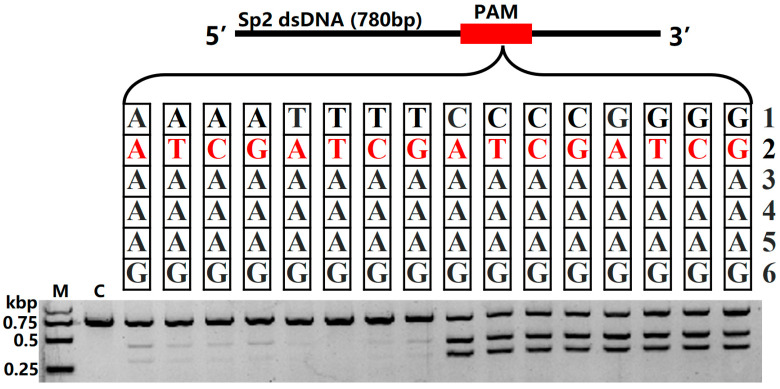
Impact of single-nucleotide substitutions in the PAM on Fp2Cas9 DNA cleavage activity (M, maker; kbp, kilobase pairs; C, control).

Building upon our previous identification of the Fp2Cas9 PAM as 5′-NNAAAG-3′ [30], we aimed to define its sequence preferences more precisely. To identify functional PAM variants and quantify their relative activities, we systematically substituted single nucleotides at the first two variable positions (N1 and N2) of the motif (Table S1). Specifically, single-nucleotide substitutions were introduced into the PAM, and the cleavage efficiency of Fp2Cas9 was assessed on target sequences containing these variants (Figure 3). Analysis revealed that Fp2Cas9 is tolerant at the second nucleotide position, indicating low sequence specificity at this site. In contrast, the first nucleotide position showed a clear preference: guanine (G) or cytosine (C) supported significantly higher cleavage activity than other nucleotides, suggesting their importance for optimal Fp2Cas9-PAM recognition or complex stability. Collectively, these results refine the Fp2Cas9 PAM requirement to 5′-SNAAAG-3′ (S = G/C).

### 2.4. Fp2Cas9 sgRNA Design

**Figure 4:**
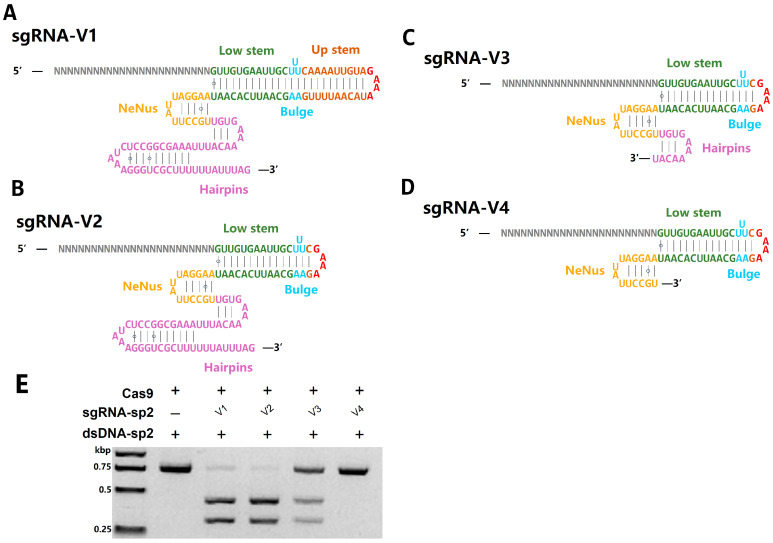
Design and functional evaluation of sgRNA variants for Fp2Cas9. (**A**) Schematic of sgRNA-V1. (**B**) Schematic of sgRNA-V2. (**C**) Schematic of sgRNA-V3. (**D**) Schematic of sgRNA-V4. (**E**) In vitro DNA cleavage assay evaluating the guiding efficiency of different sgRNA variants in Fp2Cas9-mediated DNA cleavage. (kbp, kilobase pairs).

To enable programmable DNA targeting by Fp2Cas9, we utilized the truncation results from Section 2.2 to fuse the functional crRNA (7–55 nt) and tracrRNA (nucleotides 15–99 nt) via a stable GAAA tetraloop, thereby generating the sgRNA-V1 capable of directing Cas9 to specific DNA targets (Figure 4A). Guided by the study on functional gRNA fragments [31], we further modified this design by deleting the upstream region to create a more compact sgRNA-V2 and introduced systematic truncations in the hairpin regions to produce additional variants, sgRNA-V3 and sgRNA-V4 (Figure 4B–D). These sgRNA variants were evaluated using in vitro DNA cleavage assays (Figure 4E), allowing direct comparison of their efficiency in guiding Cas9-mediated nuclease activity. The results demonstrated that sgRNA-V1, sgRNA-V2, and sgRNA-V3 could all successfully replace the native crRNA–tracrRNA duplex, with sgRNA-V1 and sgRNA-V2 exhibiting the highest guiding efficiency. Considering both functionality and compactness, sgRNA-V2 was selected as the preferred variant for subsequent experimental applications.

### 2.5. Fp2Cas9 Temperature Preferences

**Figure 5:**
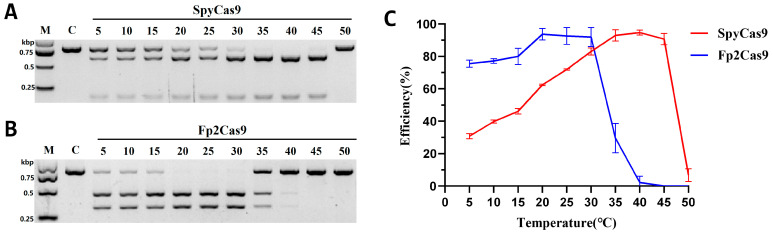
Temperature-dependent DNA cleavage activity of Fp2Cas9 compared with SpyCas9. (**A**) In vitro DNA cleavage assay of SpyCas9 at temperatures ranging from 5 to 50 °C. (**B**) In vitro DNA cleavage assay of Fp2Cas9 at temperatures ranging from 5 to 50 °C. (**C**) Quantification of DNA cleavage efficiency for SpyCas9 (red line) and Fp2Cas9 (blue line) across the temperature range of 5–50 °C. Error bars represent standard deviation from 3 independent experiments. (M, maker; kbp, kilobase pairs; C, control).

To assess the temperature-dependent cleavage activity of Fp2Cas9, SpyCas9 was used as a reference nuclease. A SpyCas9-targeting gRNA (spy-sp2-gRNA) was designed for the sp2 target using ChopChop, whereas Fp2Cas9 employed fp-sp2-gRNA-V2, as described in Section 2.4. RNP complexes (500 nM Cas9: 500 nM gRNA) were incubated with 25 nM target DNA over a temperature range of 5–50 °C. SpyCas9 showed maximal activity (above 90% substrate cleavage) at 30–40 °C, with cleavage efficiency decreasing linearly to 28% at 5 °C (Figure 5A,C). In contrast, Fp2Cas9 reached maximal nuclease activity at 20–30 °C, achieving cleavage efficiency of up to 90%. Its activity gradually decreased at lower temperatures, yet 75% cleavage efficiency was retained at 5 °C. However, Fp2Cas9 exhibited limited thermostability: cleavage efficiency declined to 29% at 35 °C and was almost completely lost at 40 °C (Figure 5B,C). These results indicate that Fp2Cas9 is a cold-adapted Cas9 nuclease with high activity at low temperatures but reduced stability at higher temperatures, highlighting its potential utility in low-temperature biological systems.

### 2.6. Nuclease Activity of Fp2Cas9 in Zebrafish Embryos

**Figure 6:**
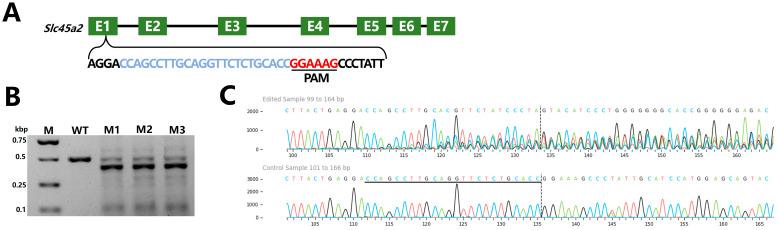
CRISPR/Fp2Cas9-mediated knockout of the zebrafish *slc45a2* gene. (**A**) Schematic representation of the zebrafish *slc45a2* gene structure and the exon 1 target site used for CRISPR/Fp2Cas9 editing. The target sequence is shown in blue, and the PAM sequence is indicated in red. (**B**) Detection of mutations in F0 zebrafish by T7EI assay. (**C**) Sanger sequencing chromatograms of F0 mutants show overlapping peaks near the target site, indicating insertions or deletions (indels). (M, DNA marker; M1–M3, F0 zebrafish injected with CRISPR/Fp2Cas9 targeting *slc45a2*; WT, wild-type control).

To evaluate the nuclease activity of Fp2Cas9 in zebrafish embryos, a recombinant Fp2Cas9 variant was engineered by fusing nuclear localization signal (NLS) peptides to both N- and C-termini of the wild-type protein. This dual-NLS modification was intended to enhance nuclear import efficiency in vivo. The expression plasmid pGEX-6P-1-2NLS-Fp2Cas9 was constructed and employed to produce the recombinant protein. Following affinity purification and buffer exchange, 2NLS-Fp2Cas9 was obtained with >95% purity, as confirmed by SDS-PAGE analysis (Figure S1). A sgRNA (fp-*slc45a2*-sgRNA1) targeting the first exon of *slc45a2* was designed according to the established 5′-SNAAAG-3′ PAM requirement (Figure 6A). Off-target analysis using ChopChop did not reveal any potential off-target sites, likely owing to the complex PAM sequence minimizing off-target risk (Figure S2). In vitro cleavage assays confirmed that 2NLS-Fp2Cas9, guided by this sgRNA, efficiently cleaved exogenous *slc45a2* substrates (Figure S3). RNP complexes were microinjected into one-cell stage embryos.

At 36 h post-fertilization(hpf), three control embryos were pooled as a single sample, and 9 injected embryos were divided into 3 separate samples for genomic DNA extraction. Target genomic regions were amplified by PCR, and indel mutations were analyzed using the T7EI assay, followed by validation via Sanger sequencing. T7EI digestion and agarose gel electrophoresis showed no cleavage bands in wild-type samples, whereas two distinct lower molecular weight bands appeared in injected embryos, indicating the presence of indel mutations in the PCR-amplified fragments (Figure 6B). Sanger sequencing showed a single distinct peak in wild-type chromatograms, while F0 mutants exhibited multiple overlapping peaks near the target site (Figure 6C), confirming successful disruption of the *slc45a2* by the CRISPR/Fp2Cas9 system.

**Figure 7:**
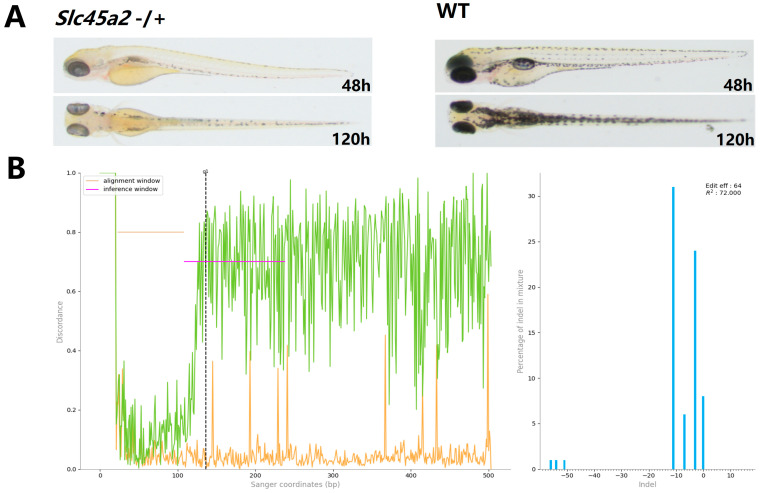
Phenotypic analysis and indel quantification in CRISPR/Fp2Cas9-edited zebrafish. (**A**) Representative phenotypic observations of mutants and control embryos at 48 and 120 hpf. Altered pigmentation is evident in F0 mutants. (**B**) ICE analysis quantifying insertion/deletion (indel) frequencies in mutant embryos at 120 hpf compared with the control. Only one representative result is shown; the other two independent samples are presented in Figure S4.

Phenotypic analysis at 48 and 120 hpf showed that 42.86% (*n* = 119) of injected embryos exhibited altered pigmentation at 48 hpf, and 43.43% (*n* = 99) displayed pigmentation changes at 120 hpf (Figure 7A). To assess mutation efficiency, three embryos showing phenotypic alterations at 120 hpf were individually selected for DNA extraction, with each processed as a separate sample. Target fragments were amplified by PCR and analyzed via Sanger sequencing. Analysis of Sanger sequencing data using ICE CRISPR analysis tool (v3.0, Synthego, CA, USA) indicated that sequence variations between injected embryos with altered pigmentation and controls were 64%, 63%, and 56%, respectively (Figure 7B and Figure S4).

## 3. Discussion

The identification and characterization of novel Cas9 orthologs with unique biochemical properties are essential for expanding the versatility of CRISPR-based genome editing. Here, we report the functional characterization of Fp2Cas9, a cold-adapted Type II-C Cas9 from *F. psychrophilum*. Our results show that Fp2Cas9 displays high DNA cleavage activity at low temperatures. Importantly, we designed a specific sgRNA that enables CRISPR/Fp2Cas9-mediated genome editing in zebrafish embryos, achieving targeted mutagenesis of the *slc45a2*. This study addresses a critical gap in CRISPR tools for cold-adapted systems and provides significant potential for genetic engineering in aquaculture and low-temperature biological systems.

The identification of the PAM sequence and the design of an adapted sgRNA scaffold represent key challenges for the downstream application of the CRISPR/Fp2Cas9 system. Bioinformatic and structural analyses confirmed that Fp2Cas9 is a Type II-C Cas9, closely related to CjCas9 and NmeCas9, both known for stringent PAM requirements and high specificity [15,32]. Building on previous studies [30], we refined the PAM preference of Fp2Cas9 to 5′-SNAAAG-3′. Although this motif is less frequent than the NGG PAM of SpCas9, its lower genomic prevalence reduces potential off-target effects [33]. Truncation experiments showed that the bulge ‘UUU’ within the crRNA is essential for activity, consistent with previous findings [31]. To optimize sgRNA design, truncations were introduced at multiple positions guided by secondary structure predictions. Deletion of the upper stem region had minimal effect, whereas truncations in the hairpin loops markedly impaired activity, suggesting that extended hairpin structures likely enhance the stability of crRNA binding to Fp2Cas9. To facilitate genome editing in zebrafish embryos, a nuclear-localized variant (2NLS-Fp2Cas9) was engineered by fusing SV40 NLS motifs to both N- and C-termini of wild-type Fp2Cas9 to improve nuclear import efficiency [34,35]. Coupling 2NLS-Fp2Cas9 with the engineered sgRNA scaffold (sgRNA-V2) enabled efficient targeted mutagenesis in zebrafish embryos. Editing of the *slc45a2* yielded a 60% insertion-deletion (indel) frequency, as quantified by ICE analysis, and 48% of injected embryos displayed a pigment-deficient phenotype at 120 hpf. These results indicate that the CRISPR/Fp2Cas9 is an effective platform for genome editing in vertebrate models.

The optimal temperature characteristics of Cas9 dictate its application scenarios. While thermophilic Cas9 variants (e.g., GeoCas9, ThermoCas9) function optimally at high temperatures (≥60 °C) [26,36], and mesophilic SpCas9 exhibits peak activity at 37 °C [37], no efficient cold-adapted Cas9 had been characterized before this study. Fp2Cas9 addresses this gap by maintaining 75% cleavage efficiency at 5 °C, which is approximately 2.5-fold higher than that of SpCas9 under the same conditions (30%). This cold adaptation is consistent with the ecological niche of *F. psychrophilum*, a pathogen of cold-water fish [38]. The molecular basis for the sustained catalytic efficiency of psychrophilic enzymes at low temperatures, relative to mesophilic or thermophilic orthologs, includes several adaptive features: a reduced number of proline and arginine residues (restricting backbone rotation and forming multiple hydrogen bonds and salt bridges, respectively), an increased number of glycine residues (enhancing local flexibility), a lower Arg/(Arg + Lys) ratio, and fewer disulfide bonds [39,40]. These compositional and structural features confer enhanced flexibility and catalytic efficiency at low temperatures [40]. In Fp2Cas9, multiple cold-adaptive traits are evident. The Arg/(Arg + Lys) ratio is relatively low (0.26), substantially lower than that of reported cold-adapted esterases such as Est97 (0.56), Est10 (0.50), and Est12 (0.33) [40,41,42]. The proline and arginine contents are only 2.65% and 4.52%, respectively. Additionally, Fp2Cas9 contains only nine cysteine residues, indicating very few intramolecular disulfide bonds, similar to Est12 [40]. It is noteworthy that in the HNH domain of Fp2Cas9, a conserved aspartate residue found in other Type II-C Cas9 homologs (e.g., D581 in AnaCas9 and D587 in Nme1Cas9) is replaced by a glutamate residue (E868) [43,44]. Although both are acidic amino acids, glutamate possesses a longer side chain, which may confer greater spatial accessibility and conformational flexibility [45]. These factors may enhance structural plasticity, thereby supporting efficient function in low-temperature catalysis, which could partially explain the cold-adaptation of Fp2Cas9.

In summary, this study reports the discovery and functional validation of the cold-adapted Fp2Cas9, providing a CRISPR tool that maintains substantial catalytic activity at low temperatures and supports precise genome editing in zebrafish. However, the relatively large size of Fp2Cas9 (1548 aa) poses challenges for vector packaging and delivery. Additionally, although its rare PAM preference may limit off-target effects, the genome-wide off-target profile across different temperatures remains to be fully assessed, as systematic cellular-level validation has not yet been performed. The limited occurrence of suitable PAM sites also constrains the applicability of this nuclease across diverse genomes. Future efforts may focus on engineering strategies such as domain truncation to reduce protein size, as well as rational design or directed evolution approaches to expand PAM compatibility to broaden its application scenarios.

## 4. Materials and Methods

### 4.1. Bioinformatical Predictions of Fp2Cas9

To characterize the function and evolutionary relationships of Fp2Cas9, a comprehensive bioinformatics analysis was performed. Amino acid sequences of representative, well-characterized Type II Cas9 orthologs were initially retrieved from public databases. Multiple sequence alignments were performed using MAFFT(v7.187, University of Tokyo, Tokyo, Japan). Ambiguously aligned regions were subsequently removed using Gblocks, followed by manual curation to ensure alignment quality. A maximum-likelihood (ML) phylogenetic tree was reconstructed from the refined alignment using RAxML (v8.1.17, Heidelberg Institute for Theoretical Studies, Heidelberg, Germany) with the GTRGAMMA substitution model. Branch support was evaluated using 1000 bootstrap pseudoreplicates. The resulting ML tree topology was visualized as a cladogram using FigTree (v1.4.0, University of Edinburgh, Edinburgh, UK). We employed AlphaFold 3 (DeepMind, London, UK) to generate structural models of the Cas9 protein in its apo form. Structural visualization and analysis were conducted using PyMOL (v3.0, DeLano Scientific LLC, New York, NY, USA).

### 4.2. Fp2Cas9 Expression and Purification

The *F. psychrophilum* CN46 Cas9 gene (GenBank: CP081493.1), codon-optimized for *E. coli* expression, was synthesized by Seven/Abcells (Harbin, China) and cloned into pUC-57 (Table S1). This gene was subsequently inserted into pGEX-6P-1 to generate pGEX-6P-1-Fp2Cas9 (Table S1). For zebrafish gene editing, SV40 nuclear localization signals (NLS) were fused to both termini of Fp2Cas9 to generate pGEX-6P-1-2NLS-Fp2Cas9 (Table S1). All proteins in this study were expressed in E. coli BL21 (DE3) competent cells and induced with 0.3 mM isopropyl-1-thio-β-D-galactopyranoside (IPTG) for 14–16 h at 18 °C. The harvested cells were resuspended in lysis buffer (25 mM Tris-HCl, pH 8.0, 1.0 M NaCl, 3 mM DTT, 1 mM PMSF) and then lysed via ultrasonication at 4 °C. The supernatant was purified with glutathione Sepharose 4B (GS4B) beads (GE Healthcare). After washing, the proteins bound to GS4B beads were released by preScission protease cleavage in buffer A (25 mM Tris-HCl, pH 8.0, 300 mM NaCl, 3 mM DTT) overnight at 4 °C and then eluted from GS4B resin. For Cas9 proteins, further fractionated by heparin sepharose column and cation exchange chromatography (GE Healthcare). Purity of the protein was assessed by denaturing 8% polyacrylamide gel electrophoresis.

### 4.3. In Vitro Transcription and Purification of RNA

RNA was synthesized in vitro with the TranscriptAid T7 High Yield Transcription Kit (Thermo Scientific, Waltham, MA, USA), using PCR-amplified DNA templates containing a T7 promoter sequence. In this study, RNAs shorter than 100 nt were isolated with TRIzol reagent (Thermo Scientific, Waltham, MA, USA), whereas those longer than 100 nt were purified with the NucleoSpin RNA Kit (Macherey-Nagel, Düren, Germany). All RNA preparations were validated by 2% agarose gel electrophoresis and spectrophotometric analysis (KAIAO, Beijing, China) before use. Complete RNA sequences are provided in Table S2.

### 4.4. In Vitro DNA Cleavage Assays

Purified tracrRNA and crRNA were annealed by heating to 95 °C for 3 min followed by gradual cooling to room temperature (~2 h). DNA cleavage assays were performed in Cas9 reaction buffer (20 mM HEPES, 150 mM KCl, 10 mM MgCl_2_, 0.5 mM DTT, 0.1 mM EDTA). Reaction mixtures (20 µL final volume) contained 1 × Cas9 reaction buffer, 500 nM Cas9, 500 nM crRNA-tracrRNA dimer (or 500 nM sgRNA), and 25 nM target dsDNA. Specifically, reactions were assembled by sequentially adding 10 × Cas9 reaction buffer, Cas9, and the crRNA:tracrRNA dimer to DEPC-treated water. After mixing, the complex was incubated at 18 °C for 10 min to allow ribonucleoprotein (RNP) formation. The target dsDNA was added and the reaction volume adjusted to 20 µL with DEPC-treated water, followed by incubation at 18 °C for 1 h to allow DNA cleavage. Reactions were terminated by adding 2 µL proteinase K and heating at 37 °C for 15 min. Cleavage products were resolved by electrophoresis on a 1.5% (*w*/*v*) agarose gel prepared in 1× TAE buffer and stained with GelStain (TransGen Biotech, Beijing, China). Electrophoresis was performed at 5–6 V/cm for 45 min. Gels were visualized using a UV transilluminator and images were captured with a gel documentation system.

For temperature-dependent cleavage assays of Fp2Cas9, SpyCas9 (New England Biolabs, MA, USA, M0386T) was included as a control enzyme. Target-specific sgRNA for SpyCas9 (spy-sp2-sgRNA), designed to cleave sp2-target DNA, was identified using the CHOPCHOP online tool (Table S2). This spy-sp2-sgRNA was synthesized via T7 in vitro transcription followed by purification. RNP were pre-assembled under enzyme-specific conditions: Fp2Cas9 RNP (500 nM Fp2Cas9 + 500 nM Fp-sgRNA-v2) in standard cleavage buffer (20 mM HEPES, 150 mM KCl, 10 mM MgCl_2_, 0.5 mM DTT, 0.1 mM EDTA, pH 7.5) at 18 °C for 10 min, while SpyCas9 RNP (500 nM SpyCas9 + 500 nM Spy-sgRNA) was prepared in NEBuffer 3.1 at 28 °C for 10 min per manufacturer protocol. To rigorously control thermal effects, pre-assembled RNPs and sp2-target dsDNA (25 nM) were independently pre-equilibrated at specified temperatures (5–50 °C) for 5 min. Reactions were initiated by combining dsDNA with RNPs, followed by 1 h incubation at target temperatures. All reactions were terminated and analyzed as described above. Cleavage efficiency was quantified using ImageJ (v1.54p) via the formula: Cleavage efficiency (%) = [Intensity of cleaved products/(Intensity of cleaved products + uncleaved substrate)] × 100%.

### 4.5. CRISPR/Fp2Cas9 Targeted Editing of Zebrafish slc45a2 and Phenotypic Analysis

The sgRNA target sites within the *slc45a2* gene was identified based on the 5′-N24SNAAAG-3′ on either the sense or antisense strand using the online tool CHOPCHOP (https://chopchop.cbu.uib.no/) accessed on 12 March 2025. Candidate target site was selected from the first exon of the *slc45a2* (gRNA sequences are provided in Table S3).

The zebrafish *slc45a2* fragment containing the sgRNA target site was amplified with specific primers (Table S2). The cleavage activity of the sgRNA on the *slc45a2* target DNA was subsequently verified using the in vitro digestion assay described in Section 4.4. After confirming sgRNA activity, the RNP complex (500 nM sgRNA and 500 nM Fp2Cas9) was co-injected with phenol red into one-cell-stage zebrafish embryos using a MPPI-3 pressure injector (ASI, Springville, UT, USA). For the control groups, only phenol red was injected into embryos at the same developmental stage.

Genomic and phenotypic assessment of mutagenesis was performed as follows. At 36 hpf, 9 experimental embryos were randomly divided into 3 groups (*n* = 3) for independent genomic DNA extraction, while 3 control embryos were pooled as a single reference sample. The target gene was amplified using the primers listed in Table S2. Mutation analysis was performed using two methods: cleavage detection through T7 endonuclease I (T7EI; New England Biolabs, M0302S) digestion of PCR products, and direct characterization of mutations at target sites via Sanger sequencing. For T7EI, PCR products were denatured at 95 °C for 5 min and reannealed via a temperature gradient: cooling to 85 °C at −2 °C/s then to 25 °C at −0.1 °C/s. Reannealed products were digested with T7EI at 37 °C for 30 min and analyzed on 1.5% agarose gels. Indels were identified by overlapping peaks in Sanger chromatograms near target sites. Phenotypic assessment was conducted at 48 and 120 hpf using a Leica stereomicroscope (Leica, Wetzlar, Germany)to document pigmentation alterations, and phenotypic mutation rates were quantified. Mutation rate (%) = (Number of embryos with pigmentation alterations/Total viable embryos) × 100. At 120 hpf, 3 individuals with confirmed phenotypic mutations were collected for genomic DNA extraction, followed by indel quantification using ICE CRISPR Analysis(v3.0, EditCo Bio, Redwood City, CA, USA).

## 5. Conclusions

In this study, we characterized Fp2Cas9, a cold-adapted Type II-C nuclease from *F. psychrophilum*. Fp2Cas9 preferentially recognizes the PAM sequence 5′-SNAAAG-3′, retains efficient DNA cleavage activity at 5 °C, and shows a marked reduction in activity at 35 °C. The development of a compact sgRNA scaffold enabled efficient programmable targeting, and in vivo experiments in zebrafish confirmed its capacity to introduce mutations and generate visible phenotypes. Overall, these findings indicate that Fp2Cas9 functions effectively as a genome-editing nuclease under cold conditions and may serve as a useful tool for genetic studies and applications in cold-water organisms and other low-temperature systems.

## Data Availability

Data is contained within the article.

## Supplementary Materials

The following supporting information can be downloaded at: https://www.mdpi.com/article/10.3390/ijms262110681/s1.

## Abbreviations

The following abbreviations are used in this manuscript:

CRISPR	Clustered Regularly Interspaced Short Palindromic Repeats
PAM	Protospacer Adjacent Motif
sgRNA	single-guide RNA
RNP	Ribonucleoprotein
NLS	Nuclear Localization Signal
hpf	hours post-fertilization

## References

[1] Marraffini L.A., Sontheimer E.J. (2008). CRISPR interference limits horizontal gene transfer in staphylococci by targeting DNA. Science.

[2] Marraffini L.A. (2015). CRISPR-Cas immunity in prokaryotes. Nature.

[3] Sontheimer E.J., Barrangou R. (2015). The Bacterial Origins of the CRISPR Genome-Editing Revolution. Hum. Gene Ther..

[4] Barrangou R., Fremaux C., Deveau H., Richards M., Boyaval P., Moineau S., Romero D.A., Horvath P. (2007). CRISPR Provides Acquired Resistance Against Viruses in Prokaryotes. Science.

[5] Brouns S.J., Jore M.M., Lundgren M., Westra E.R., Slijkhuis R.J., Snijders A.P., Dickman M.J., Makarova K.S., Koonin E.V., van der Oost J. (2008). Small CRISPR RNAs guide antiviral defense in prokaryotes. Science.

[6] Mohanraju P., Makarova K.S., Zetsche B., Zhang F., Koonin E.V., van der Oost J. (2016). Diverse evolutionary roots and mechanistic variations of the CRISPR-Cas systems. Science.

[7] Makarova K.S., Wolf Y.I., Alkhnbashi O.S., Costa F., Shah S.A., Saunders S.J., Barrangou R., Brouns S.J., Charpentier E., Haft D.H. (2015). An updated evolutionary classification of CRISPR-Cas systems. Nat. Rev. Microbiol..

[8] Chylinski K., Makarova K.S., Charpentier E., Koonin E.V. (2014). Classification and evolution of type II CRISPR-Cas systems. Nucleic. Acids. Res..

[9] Jinek M., Chylinski K., Fonfara I., Hauer M., Doudna J.A., Charpentier E. (2012). A programmable dual-RNA-guided DNA endonuclease in adaptive bacterial immunity. Science.

[10] Cong L., Ran F.A., Cox D., Lin S., Barretto R., Habib N., Hsu P.D., Wu X., Jiang W., Marraffini L.A. (2013). Multiplex genome engineering using CRISPR/Cas systems. Science.

[11] Shmakov S., Smargon A., Scott D., Cox D., Pyzocha N., Yan W., Abudayyeh O.O., Gootenberg J.S., Makarova K.S., Wolf Y.I. (2017). Diversity and evolution of class 2 CRISPR-Cas systems. Nat. Rev. Microbiol..

[12] Wright A.V., Nuñez J.K., Doudna J.A. (2016). Biology and Applications of CRISPR Systems: Harnessing Nature’s Toolbox for Genome Engineering. Cell.

[13] Selkova P., Vasileva A., Arseniev A., Abramova M., Musharova O., Malysheva P., Khodorkovskii M., Severinov K. (2023). Characterization of Streptococcus uberis Cas9 (SuCas9)—A Type II-A Ortholog Functional in Human Cells. BioRxiv.

[14] Ran F.A., Cong L., Yan W.X., Scott D.A., Gootenberg J.S., Kriz A.J., Zetsche B., Shalem O., Wu X., Makarova K.S. (2015). In vivo genome editing using Staphylococcus aureus Cas9. Nature.

[15] Kim E., Koo T., Park S.W., Kim D., Kim K., Cho H.-Y., Song D.W., Lee K.J., Jung M.H., Kim S. (2017). In vivo genome editing with a small Cas9 orthologue derived from Campylobacter jejuni. Nat. Commun..

[16] Hou Z., Zhang Y., Propson N.E., Howden S.E., Chu L.F., Sontheimer E.J., Thomson J.A. (2013). Efficient genome engineering in human pluripotent stem cells using Cas9 from Neisseria meningitidis. Proc. Natl. Acad. Sci. USA.

[17] Matveeva A., Ryabchenko A., Petrova V., Prokhorova D., Zhuravlev E., Zakabunin A., Tikunov A., Stepanov G. (2023). 17Expression and Functional Analysis of the Compact Thermophilic Anoxybacillus flavithermus Cas9 Nuclease. IJMS.

[18] Zhang Y., Zhang H., Xu X., Wang Y., Chen W., Wang Y., Wu Z., Tang N., Wang Y., Zhao S. (2020). Catalytic-state structure and engineering of Streptococcus thermophilus Cas9. Nat. Catal..

[19] Mir A., Edraki A., Lee J., Sontheimer E.J. (2018). Type II-C CRISPR-Cas9 Biology, Mechanism, and Application. ACS Chem. Biol..

[20] Xu R., Qin R., Xie H., Li J., Liu X., Zhu M., Sun Y., Yu Y., Lu P., Wei P. (2022). Genome editing with type II-C CRISPR-Cas9 systems from Neisseria meningitidis in rice. Plant Biotechnol. J..

[21] Lin L., Petersen T.S., Jensen K.T., Bolund L., Kühn R., Luo Y. (2017). Fusion of SpCas9 to E. coli Rec A protein enhances CRISPR-Cas9 mediated gene knockout in mammalian cells. J. Biotechnol..

[22] Bauer D.E., Canver M.C., Orkin S.H. (2015). Generation of genomic deletions in mammalian cell lines via CRISPR/Cas9. J. Vis. Exp..

[23] Hsu P.D., Scott D.A., Weinstein J.A., Ran F.A., Konermann S., Agarwala V., Li Y., Fine E.J., Wu X., Shalem O. (2013). DNA targeting specificity of RNA-guided Cas9 nucleases. Nat. Biotechnol..

[24] Kurokawa S., Rahman H., Yamanaka N., Ishizaki C., Islam S., Aiso T., Hirata S., Yamamoto M., Kobayashi K., Kaya H. (2021). A Simple Heat Treatment Increases SpCas9-Mediated Mutation Efficiency in Arabidopsis. Plant Cell Physiol..

[25] Harrington L.B., Paez-Espino D., Staahl B.T., Chen J.S., Ma E., Kyrpides N.C., Doudna J.A. (2017). A thermostable Cas9 with increased lifetime in human plasma. Nat. Commun..

[26] Mougiakos I., Mohanraju P., Bosma E.F., Vrouwe V., Finger Bou M., Naduthodi M.I.S., Gussak A., Brinkman R.B.L., van Kranenburg R., van der Oost J. (2017). Characterizing a thermostable Cas9 for bacterial genome editing and silencing. Nat. Commun..

[27] Handeland S.O., Björnsson, Arnesen A.M., Stefansson S.O. (2003). Seawater adaptation and growth of post-smolt Atlantic salmon (Salmo salar) of wild and farmed strains. Aquaculture.

[28] Jiang X., Dong S., Liu R., Huang M., Dong K., Ge J., Gao Q., Zhou Y. (2021). Effects of temperature, dissolved oxygen, and their interaction on the growth performance and condition of rainbow trout (Oncorhynchus mykiss). J. Therm. Biol..

[29] Xiang G., Zhang X., An C., Cheng C., Wang H. (2017). Temperature effect on CRISPR-Cas9 mediated genome editing. J. Genet. Genom..

[30] Chen F., Wang D., Lu T., Li S. (2023). Identification of a novel type II-C Cas9 from the fish pathogen *Flavobacterium psychrophilum*. Front. Microbiol..

[31] Briner A.E., Donohoue P.D., Gomaa A.A., Selle K., Slorach E.M., Nye C.H., Haurwitz R.E., Beisel C.L., May A.P., Barrangou R. (2014). Guide RNA Functional Modules Direct Cas9 Activity and Orthogonality. Mol. Cell.

[32] Amrani N., Gao X.D., Liu P., Edraki A., Mir A., Ibraheim R., Gupta A., Sasaki K.E., Wu T., Donohoue P.D. (2018). NmeCas9 is an intrinsically high-fidelity genome-editing platform. Genome Biol..

[33] Mengstie M.A., Azezew M.T., Dejenie T.A., Teshome A.A., Admasu F.T., Teklemariam A.B., Mulu A.T., Agidew M.M., Adugna D.G., Geremew H. (2024). Recent Advancements in Reducing the Off-Target Effect of CRISPR-Cas9 Genome Editing. Biol. Targets Ther..

[34] Shinkado S., Saito H., Yamazaki M., Kotera S., Arazoe T., Arie T., Kamakura T. (2022). Genome editing using a versatile vector-based CRISPR/Cas9 system in Fusarium species. Sci. Rep..

[35] Hu P., Zhao X., Zhang Q., Li W., Zu Y. (2018). Comparison of Various Nuclear Localization Signal-Fused Cas9 Proteins and Cas9 mRNA for Genome Editing in Zebrafish. G3 Genes|Genomes|Genet..

[36] Belato H.B., Knight A.L., Alexandra M.D., Pindi C., Fan Z., Luo J., Palermo G., Jogl G., Lisi G.P. (2025). Structural and dynamic impacts of single-atom disruptions to guide RNA interactions within the recognition lobe of Geobacillus stearothermophilus Cas9. eLife.

[37] Montecillo J.A.V., Chu L.L., Bae H. (2020). CRISPR-Cas9 System for Plant Genome Editing: Current Approaches and Emerging Developments. Agronomy.

[38] Li S., Chai J., Knupp C., Nicolas P., Wang D., Cao Y., Deng F., Chen F., Lu T., Loch T.P. (2021). Phenotypic and Genetic Characterization of *Flavobacterium psychrophilum* Recovered from Diseased Salmonids in China. Microbiol. Spectr..

[39] Cavicchioli R., Siddiqui K.S., Andrews D., Sowers K.R. (2002). Low-temperature extremophiles and their applications. Curr. Opin. Biotechnol..

[40] Wu G., Zhang S., Zhang H., Zhang S., Liu Z. (2013). A novel esterase from a psychrotrophic bacterium *Psychrobacter celer* 3Pb1 showed cold-adaptation and salt-tolerance. J. Mol. Catal. B Enzym..

[41] Fu J., Leiros H.-K.S., de Pascale D., Johnson K.A., Blencke H.-M., Landfald B. (2013). Functional and structural studies of a novel cold-adapted esterase from an Arctic intertidal metagenomic library. Appl. Microbiol. Biotechnol..

[42] Wu G., Wu G., Zhan T., Shao Z., Liu Z. (2013). Characterization of a cold-adapted and salt-tolerant esterase from a psychrotrophic bacterium Psychrobacter pacificensis. Extremophiles.

[43] Jinek M., Jiang F., Taylor D.W., Sternberg S.H., Kaya E., Ma E., Anders C., Hauer M., Zhou K., Lin S. (2014). Structures of Cas9 Endonucleases Reveal RNA-Mediated Conformational Activation. Science.

[44] Sun W., Yang J., Cheng Z., Amrani N., Liu C., Wang K., Ibraheim R., Edraki A., Huang X., Wang M. (2019). Structures of Neisseria meningitidis Cas9 Complexes in Catalytically Poised and Anti-CRISPR-Inhibited States. Mol. Cell.

[45] Tolmachev D., Mamistvalov G., Lukasheva N., Larin S., Karttunen M. (2021). Effects of Amino Acid Side-Chain Length and Chemical Structure on Anionic Polyglutamic and Polyaspartic Acid Cellulose-Based Polyelectrolyte Brushes. Polymers.

