# Supplementary material for: Functional Characterization of Fp2Cas9, a Cold-Adapted Type II-C CRISPR Nuclease from Flavobacterium psychrophilum
Source: Int J Mol Sci. 2025 Nov 2;26(21):10681. doi: 10.3390/ijms262110681 (PMC12609912; doi:10.3390/ijms262110681)
Supplement: Supplementary file 1 [file ijms-26-10681-s001.zip › ijms-3929515-supplementary.pdf]

Supplementary Table S1.DNA sequences used in this study.

Plasmids	Relevant characteristics	Source
**Plasmids for Cas9 expression**		
pUC57- Fp2Cas9	pUC57 cloning vector for codon-optimized *F. psychrophilum* Fp2Cas9	This study
pGEX-6P-1-Fp2Cas9	pGEX-6P-1 expression vector for *F. psychrophilum* Fp2Cas9	This study
pGEX-6P-1-2NLSFp2Cas9	pGEX-6P-1 expression vector for NLS-Fp2Cas9-NLS	This study
**Plasmids for protospacer cleavage assays**		
pUC57-CN46-spacer2	pUC57 with CN46protospacer 2 and WT PAM(CAAAAG)	This study
pUC57-CN46-spacer2(AA)	pUC57 with CN46protospacer 2 and AAAAAG PAM	This study
pUC57-CN46-spacer2(AT)	pUC57 with CN46protospacer 2 and ATAAAG PAM	This study
pUC57-CN46-spacer2(AC)	pUC57 with CN46protospacer 2 and ACAAAG PAM	This study
pUC57-CN46-spacer2(AG)	pUC57 with CN46protospacer 2 and AGAAAG PAM	This study
pUC57-CN46-spacer2(TA)	pUC57 with CN46protospacer 2 and TAAAAG PAM	This study
pUC57-CN46-spacer2(TT)	pUC57 with CN46protospacer 2 and TTAAAG PAM	This study
pUC57-CN46-spacer2(TC)	pUC57 with CN46protospacer 2 and TCAAAG PAM	This study
pUC57-CN46-spacer2(TG)	pUC57 with CN46protospacer 2 and TGAAAG PAM	This study
pUC57-CN46-spacer2(CT)	pUC57 with CN46protospacer 2 and CTAAAG PAM	This study
pUC57-CN46-spacer2(CC)	pUC57 with CN46protospacer 2 and CCAAAG PAM	This study
pUC57-CN46-spacer2(CG)	pUC57 with CN46protospacer 2 and CGAAAG PAM	This study
pUC57-CN46-spacer2(GA)	pUC57 with CN46protospacer 2 and GAAAAG PAM	This study
pUC57-CN46-spacer2(GT)	pUC57 with CN46protospacer 2 and GTAAAG PAM	This study
pUC57-CN46-spacer2(GC)	pUC57 with CN46protospacer 2 and GCAAAG PAM	This study
pUC57-CN46-spacer2(GG)	pUC57 with CN46protospacer 2 and GGAAAG PAM	This study

Supplementary Table S2. RNA sequences used in this study.

Purpose	Sequence 5'-3'
tracrRNA (1-99nt)	GGUUGUAGUAUUGUACUACAAUUUUGAAGCAAUUCACAAUAAGGAUUAUUCCGUUGUGAAAACAUUUAAAGCGGCCUCUAAAGGGUCGCUUUUUUAUUUAG
crRNA-sp2(1-55nt)	GGACCAAUUUUUGAUACAUCGUAUUAAAUCGCGUUGUGAAUUGCUUUCAAAAUUGUA
crRNA-sp2(1-50nt)	GGACCAAUUUUUGAUACAUCGUAUUAAAUCGCGUUGUGAAUUGCUUUCAAAA
crRNA-sp2(1-46nt)	GGACCAAUUUUUGAUACAUCGUAUUAAAUCGCGUUGUGAAUUGCUUUC
crRNA-sp2(1-44nt)	GGACCAAUUUUUGAUACAUCGUAUUAAAUCGCGUUGUGAAUUGCUU
tracrRNA(15-99nt)	GGUACAAUUUUGAAGCAAUUCACAAUAAGGAUUAUUCCGUUGUGAAAACAUUUAAAGCGGCCUCUAAAGGGUCGCUUUUUUAUUUAG
tracrRNA(25-99nt)	GGAAGCAAUUCACAAUAAGGAUUAUUCCGUUGUGAAAACAUUUAAAGCGGCCUCUAAAGGGUCGCUUUUUUAUUUAG
tracrRNA(27-99nt)	GGGCAAUUCACAAUAAGGAUUAUUCCGUUGUGAAAACAUUUAAAGCGGCCUCUAAAGGGUCGCUUUUUUAUUUAG
tracrRNA(25-93nt)	GGAAGCAAUUCACAAUAAGGAUUAUUCCGUUGUGAAAACAUUUAAAGCGGCCUCUAAAGGGUCGCUUUUUU
tracrRNA(25-64nt)	GGAAGCAAUUCACAAUAAGGAUUAUUCCGUUGUGAAAACAUUU
tracrRNA(25-52nt)	GGAAGCAAUUCACAAUAAGGAUUAUUCCGU
crRNA-sp2(3-55nt)	GGCAAUUUUUGAUACAUCGUAUUAAAUCGCGUUGUGAAUUGCUUUCAAAAUUGUA
crRNA-sp2(5-55nt)	GGAUUUUUGAUACAUCGUAUUAAAUCGCGUUGUGAAUUGCUUUCAAAAUUGUA
crRNA-sp2(7-55nt)	GGUUUUGAUACAUCGUAUUAAAUCGCGUUGUGAAUUGCUUUCAAAAUUGUA
crRNA-sp2(9-55nt)	GGUUGAUACAUCGUAUUAAAUCGCGUUGUGAAUUGCUUUCAAAAUUGUA
crRNA-sp2(11-55nt)	GGGAUACAUCGUAUUAAAUCGCGUUGUGAAUUGCUUUCAAAAUUGUA
crRNA-sp2(13-55nt)	GGUACAUCGUAUUAAAUCGCGUUGUGAAUUGCUUUCAAAAUUGUA
Fp-sp2-sgRNA-V1	GGUUUUGAUACAUCGUAUUAAAUCGCGUUGUGAAUUGCUUUCAAAAUUGUAGAAAUACAAUUUUGAAGCAAUUCACAAUAAGGAUUAUUCCGUUGUGAAAACAUU

	UAAAGCGGCCUCUAAAGGGUCGCUUUUUUAUUUAG
Fp-sp2-sgRNA-V2	GGUUUUGAUACAUCGUAUUAAAUCGCGUUGUGAAUUGCUUUCGAAAGAAGCAAUUCACAAUAAGGAUUAUUCCGUUGUGAAAACAUUUAAAGCGGCCUCUAAAGGGUCGCUUUUUUAUUUAG
Fp-sp2-sgRNA-V3	GGUUUUGAUACAUCGUAUUAAAUCGCGUUGUGAAUUGCUUUCGAAAGAAGCAAUUCACAAUAAGGAUUAUUCCGUUGUGAAAACAU
Fp-sp2-sgRNA-V4	GGUUUUGAUACAUCGUAUUAAAUCGCGUUGUGAAUUGCUUUCGAAAGAAGCAAUUCACAAUAAGGAUUAUUCCGU
Spy-sp2-sgRNA	GGUACACCAACAUAUAGCAGCUGUUUUAGAGCUAGAAAUAGCAAGUUAAAAUAAGGCUAGUCCGUUAUCAACUUGAAAAAGUGGCACCGAGUCGGUGCUUUUCACAA
fp-*slc45a2*-sgRNA1	GGCCAGCCUUGCAGGUUCUCUGCACCGUUGUGAAUUGCUUUCGAAAGAAGCAAUUCACAAUAAGGAUUAUUCCGUUGUGAAAACAUUUAAAGCGGCCUCUAAAGGGUCGCUUUUUUAUUUAG

Supplementary Table S3. Primers used in this study.

Primers	Sequence 5'-3'	Relevant characteristics
CN46Sp2-F	TCCATTTTTTCCAGTAGTTTGTATAA	For amplification of the spacer2 template in *in vitro* cleavage assays.
CN46Sp2-R	TTTTATATCGTTAAATATAGAGGT	For amplification of the spacer2 template in *in vitro* cleavage assays.
Slc45a2-F	CACATGCTTACAAGCCGAGC	For amplification of the *slc45a2* template in *in vitro* cleavage assays.
Slc45a2-R	GTGACTGCATCTCCATTTAG	For amplification of the *slc45a2* template in *in vitro* cleavage assays.

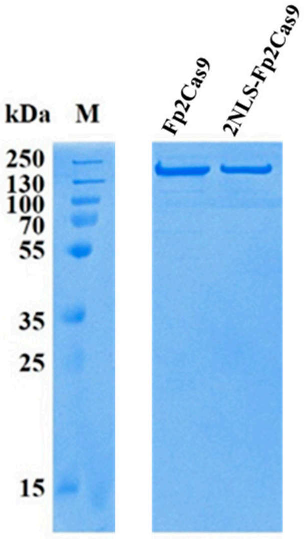

Figure S1. An SDS gel showing purified recombinant Fp2Cas9 and 2NLS-Fp2Cas9.

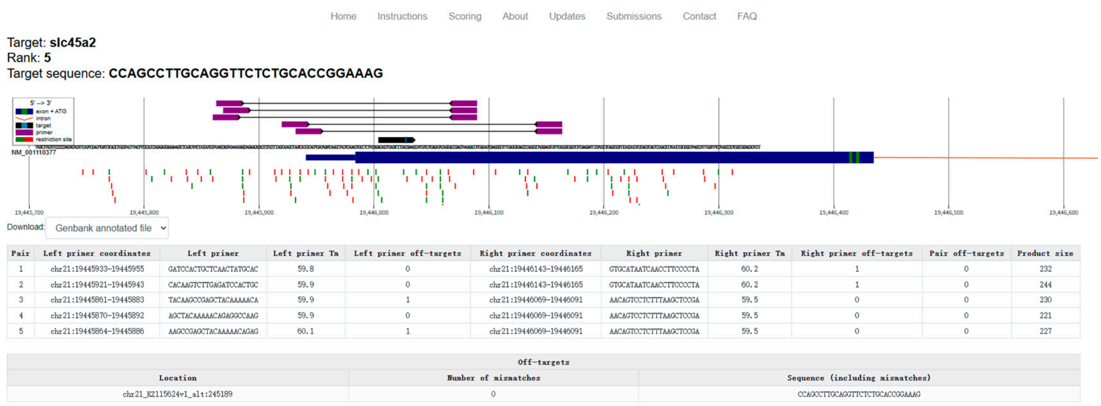

Pair	Left primer coordinates	Left primer	Left primer Tm	Left primer off-targets	Right primer coordinates	Right primer	Right primer Tm	Right primer off-targets	Pair off-targets	Product size
1	chr21:19445933-19445955	GATCCACTGCTCAACTATGCAC	59.8	0	chr21:19446143-19446165	GTGCATAATCAACCTTCCCCTA	60.2	1	0	232
2	chr21:19445921-19445943	CACAAGTCTTGAGATCCACTGC	59.9	0	chr21:19446143-19446165	GTGCATAATCAACCTTCCCCTA	60.2	1	0	244
3	chr21:19445861-19445883	TACAAGCCGAGCTACAAAAACA	59.9	1	chr21:19446069-19446091	AACAGTCCTCTTTAAGCTCCGA	59.5	0	0	230
4	chr21:19445870-19445892	AGCTACAAAAACAGAGGCCAAG	59.9	0	chr21:19446069-19446091	AACAGTCCTCTTTAAGCTCCGA	59.5	0	0	221
5	chr21:19445864-19445886	AAGCCGAGCTACAAAAACAGAG	60.1	1	chr21:19446069-19446091	AACAGTCCTCTTTAAGCTCCGA	59.5	0	0	227

Off-targets		
Location	Number of mismatches	Sequence (including mismatches)
chr21_KZ115624v1_alt:245189	0	CCAGCCTTGCAGGTTCTCTGCACCGGAAAG

Figure S2. ChopChop analysis of Fp-*slc45a2*-sgRNA

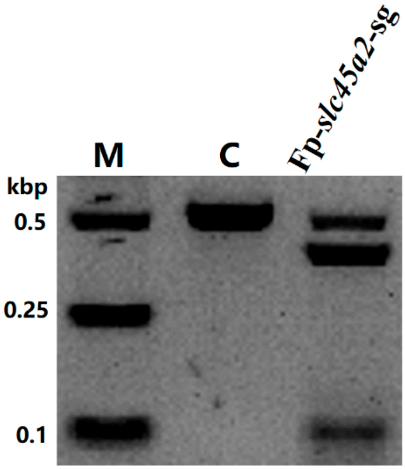

Figure S3. Fp-slc45a2-sgRNA-directed cleavage of *slc45a2* by 2NLS-Fp2Cas9 in vitro.

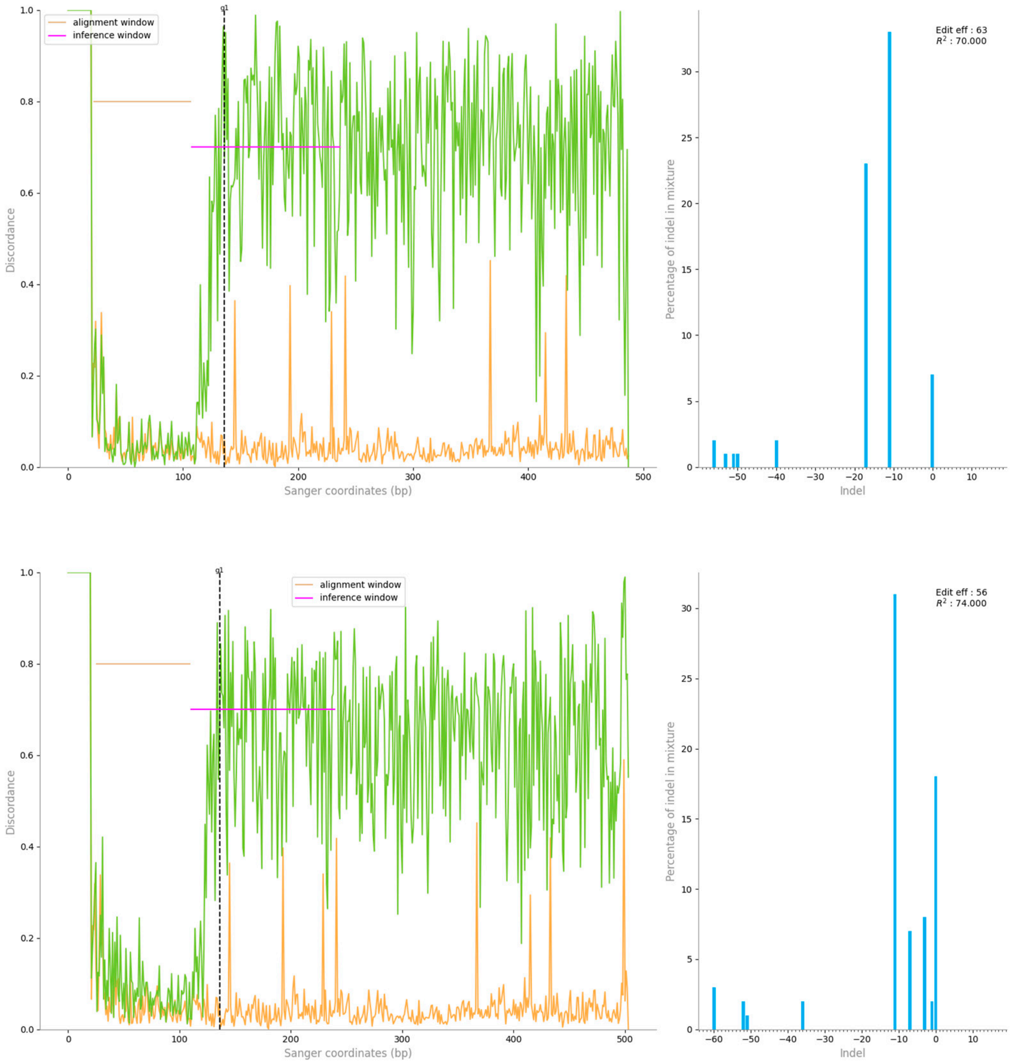

Figure S4. ICE analysis quantifying insertion/deletion (indel) frequencies in mutant embryos at 120 hpf compared with the control.
